# Free Fatty Acid Receptors as Mediators and Therapeutic Targets in Liver Disease

**DOI:** 10.3389/fphys.2021.656441

**Published:** 2021-04-07

**Authors:** Jordan D. Secor, Scott C. Fligor, Savas T. Tsikis, Lumeng J. Yu, Mark Puder

**Affiliations:** Vascular Biology Program and Department of Surgery, Boston Children’s Hospital, Harvard Medical School, Boston, MA, United States

**Keywords:** free fatty acid receptor, fatty acid, G protein-coupled receptor, liver disease, non-alcoholic fatty liver disease, non-alcoholic steatohepatitis, intestinal failure-associated liver disease

## Abstract

Free fatty acid receptors (FFARs) are a class of G protein-coupled receptors (GPCRs) that have wide-ranging effects on human physiology. The four well-characterized FFARs are FFAR1/GPR40, FFAR2/GPR43, FFAR3/GPR41, and FFAR4/GPR120. Short-chain (<6 carbon) fatty acids target FFAR2/GPR43 and FFAR3/GPR41. Medium- and long-chain fatty acids (6–12 and 13–21 carbon, respectively) target both FFAR1/GPR40 and FFAR4/GPR120. Signaling through FFARs has been implicated in non-alcoholic fatty liver disease (NAFLD), non-alcoholic steatohepatitis (NASH), intestinal failure-associated liver disease (IFALD), and a variety of other liver disorders. FFARs are now regarded as targets for therapeutic intervention for liver disease, diabetes, obesity, hyperlipidemia, and metabolic syndrome. In this review, we provide an in-depth, focused summary of the role FFARs play in liver health and disease.

## Introduction

According to the WHO, in 2016 nearly 40% of adults worldwide were overweight and, of those, 13% were obese. Type 2 diabetes mellitus (T2DM) and non-alcoholic fatty liver disease (NAFLD) are obesity-related conditions that continue to increase in prevalence as the global obesity epidemic worsens (Mantovani et al., 2020). Free fatty acid receptors (FFARs) are a previously orphan class of G protein-coupled receptors (GPCRs) that are now understood to mediate metabolic signaling effects in response to fatty acid (FA) agonism. Obesity, T2DM, and NAFLD are an intimately related set of conditions that each contribute to the metabolic syndrome phenotype (Lusis et al., 2008). The effects of FFAR signaling on these conditions and the potential for therapeutic intervention through these receptors have only recently been investigated.

**Figure 1:**
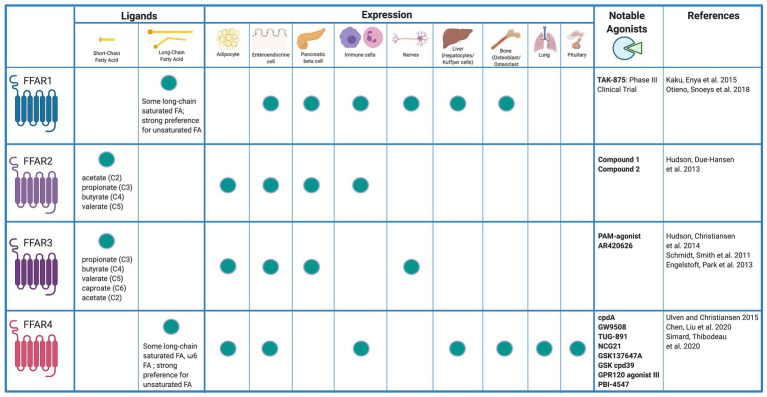
Free fatty acid receptor distribution and agonists. *Created with* *Biorender.com*.

**Figure 2:**
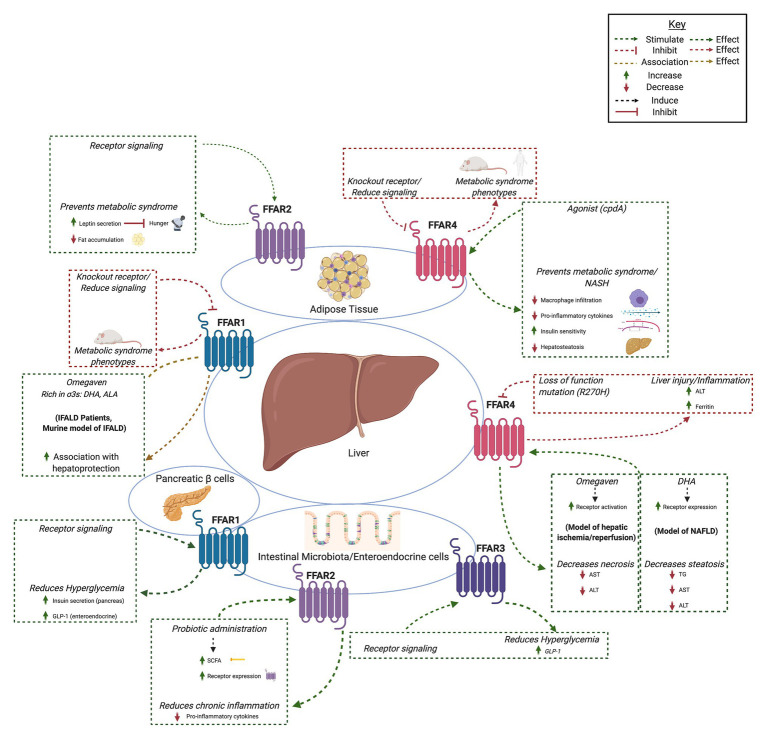
Summary of hepatic effects of free fatty acid receptor (FFAR) signaling. FFAR1-4 each stimulates complex signaling pathways that affect gastrointestinal hormone secretion, carbohydrate and lipid metabolism, adipocyte differentiation, and immunomodulation. Common themes include FFAR knockout and antagonism associated with metabolic syndrome and liver injury (red boxes and arrows) and increased FFAR expression, signaling, or agonism associated with hepatoprotection, decreased hyperglycemia, and anti-inflammation (green boxes and arrows). *Created with* *Biorender.com*.

Fatty acids are liberated by hydrolysis of dietary triacylglycerol into glycerol and FAs in the digestive tract. FAs are categorized by length and the presence or absence of carbon–carbon double bonds (i.e., saturation). While some degree of overlap exists, each FFAR is characterized by a unique combination of tissue-specific expression, FA agonist affinity, and signaling and metabolic effects (Figure 1). The physiologic interplay between obesity, T2DM, and NAFLD centers FFARs as prime therapeutic targets for these conditions. Furthermore, intestinal failure-associated liver disease (IFALD) and other metabolic liver disorders may also benefit from FFAR-targeted therapies. Here, we systematically categorize each FFAR in terms of cell and tissue distribution, affected signaling pathways, pathophysiology, and receptor agonism as they relate to opportunities for intervention in liver disease (Figure 2).

## FFAR1/GPR40

FFAR1 is a 300 amino acid, 31.45 kDa membrane protein encoded by the single exon FFAR1 gene on chromosome 19. FFAR1 is highly expressed in insulin-producing pancreatic β cells, enteroendocrine K and L cells, immune cells, taste buds, and the central nervous system (Kimura et al., 2020). To a lesser degree, FFAR1 is expressed in pancreatic *α* cells, enteroendocrine I cells, and osteoblasts and osteoclasts (Mancini and Poitout, 2013). Newer evidence suggests that FFAR1 is also expressed in hepatocytes (Lu et al., 2021). Medium chain fatty acids (MCFAs) and long chain fatty acids (LCFAs) serve as agonists at FFAR1 with LCFAs exhibiting greater potency (Briscoe et al., 2003). Saturated and unsaturated FAs both exhibit high FFAR1 potency with micromolar range minimum effective concentrations *in vitro* (Christiansen et al., 2015).

Activation of FFAR1 in pancreatic beta cells results in Gq signaling and subsequent potentiation of glucose-stimulated insulin secretion (GSIS; Itoh et al., 2003). In enteroendocrine I, K, and L cells, FFAR1 agonism results in Gs signaling and elaboration of the hormones cholecystokinin (CCK), glucose-dependent insulinotropic peptide (GIP), and glucagon-like-peptide 1 (GLP-1), respectively (Luo et al., 2012). In immune cells, specifically monocytes and hepatic Kupffer cells, FFAR1 activation leads to beta arrestin recruitment and subsequent induction of differentiation into M2 macrophages (Ohue-Kitano et al., 2018). The signaling effects of FFAR1 agonism vary widely and are tissue and agonist dependent. An understanding of the importance of FFAR1 signaling in human physiology and the potential as a therapeutic target have been attained largely through the use of knockout models and agonist/antagonist screening projects. Here we focus on FFAR1 in liver disease.

Contrary to earlier findings from Steneberg et al., FFAR1KO mice demonstrate obesity, hyperinsulinemia, and hepatic steatosis on a low-fat diet (LFD) whereas wild type mice only develop these findings on a high-fat diet (HFD; Steneberg et al., 2005; Lu et al., 2021). Furthermore, FFAR1 antagonism results in loss of the antilipogenic effects of docosahexaenoic acid (DHA) administration in cultured hepatocytes (On et al., 2019). Additionally, FFAR1KO animals exhibit diabetic, inflammatory, and obesity phenotypes which are associated with NAFLD (Kebede et al., 2008). Collectively, these findings suggest the absence of FFAR1 signaling, whether through genetic knockout or pharmacologic antagonism, results in physiology similar to that seen in patients with metabolic syndrome.

FFAR1-specific agonists have not been studied in liver disease. However, omega-3 FAs are among the most potent FFAR1 agonists, are highly concentrated in fish, seafood, nuts, and plant oils, and have been used successfully in treating liver disease. Supplementation with omega-3 FAs has shown benefit in treating patients with NAFLD, non-alcoholic steatohepatitis (NASH), alcoholic cirrhosis, and IFALD (Jump et al., 2015; Nandivada et al., 2015; Wang et al., 2019). In patients with NAFLD, omega-3 FA supplementation is associated with an increased hepatic omega 3 to omega 6 FA ratio, which in turn is associated with decreased progression of NAFLD to NASH and hepatocellular carcinoma (Puri et al., 2007). In alcoholic cirrhosis, experimental evidence suggests that omega-3 FAs may prevent ethanol-induced hepatitis and steatosis (Wada et al., 2008). IFALD is hepatic dysfunction that occurs in patients with prolonged parenteral nutrition dependence. Provision of an intravenous omega-3 fatty acid rich fish oil lipid emulsion (Omegaven®, Fresenius Kabi, United States) has contributed to the reduction in liver transplantation and mortality for pediatric IFALD patients (Gura et al., 2020).

Omega-3 FAs are potent agonists of FFAR1 and omega-3 FA supplementation is associated with improvement in NAFLD, NASH, and IFALD. However, further studies are needed to establish if omega-3 FA-induced hepatoprotection is mediated through FFAR1. Experimental models of these liver diseases used in conjunction with FFAR1 knockout animals and/or FFAR1 antagonists offer the potential to further elucidate the disease mechanisms and better characterize FFAR1 as a target for therapeutic intervention with omega-3 FAs or FFAR1-specific agonists.

A number of synthetic FFAR1 agonists have been evaluated in clinical trials treating obesity and diabetes but none are yet FDA-approved for use (Ichimura et al., 2014). Notable among these agents is the FFAR1 agonist TAK-875. In a phase III trial of treating Japanese patients with type 2 diabetes, TAK-875 was well-tolerated and demonstrated efficacy in improving glycemic control but drug development was terminated due to asymptomatic elevations in liver enzymes (Kaku et al., 2015). Subsequent toxicology studies have shown that TAK-875-induced liver injury may be mediated through formation of hepatic acyl glucuronide metabolites (Otieno et al., 2018). Currently this toxicity appears limited to TAK-875 and the implications of these findings on development of other FFAR1 agonist remains to be seen.

Many other FFAR-1 agonists are in varying stages of drug development primarily with the intention of treating type 2 diabetes (Kimura et al., 2020). To our knowledge, synthetic FFAR1 agonists have not yet been evaluated as therapies for treating metabolic liver disease in the animal models or clinical trials. Such studies would likely offer greater insight into the promising role FFAR1 plays in NAFLD, NASH, IFALD, and other liver diseases as well as potentially identify new treatments for these diseases.

## FFAR2/GPR43

FFAR2 is widely expressed in adipocytes, enteroendocrine, pancreatic β, and various inflammatory cells such as macrophages and neutrophils (Ge et al., 2008; Tolhurst et al., 2012; McNelis et al., 2015; Kamp et al., 2016). Hepatic expression of FFAR2 has not been reported. FFAR2 preferentially binds the short chain FAs (SCFAs) acetate and propionate, followed by butyrate (Brown et al., 2003; Le Poul et al., 2003). FFAR2 activation leads to intracellular coupling with the pertussis toxin-sensitive Gi/o protein and with Gq proteins (Brown et al., 2003). Activation of the Gi/o family of G proteins inhibits cAMP production and activates the ERK cascade, whereas signaling *via* Gq proteins increases intracellular calcium and promotes activation of the MAP cascade (Kimura et al., 2020). These interactions result in downstream signaling that is implicated in a wide array of metabolic effects.

FFAR2 regulates lipid metabolism and glucose levels *via* effects on hormone secretion and inflammation (Ge et al., 2008; Tolhurst et al., 2012). While not expressed in hepatocytes, FFAR2-mediated modulation of the intestinal microbiota inflammasome may have implications for NAFLD/NASH progression (Henao-Mejia et al., 2012). Oral administration of probiotics ameliorated HFD-induced NAFLD in rats *via* increased generation of SCFAs and increased FFAR2 expression (Liang, Liang et al., 2019). This effect may be due to the role of the FFAR2 pathway in inhibiting inflammatory cytokines and reducing chronic inflammation, both with positive consequences on metabolic liver disease (Liang et al., 2019). SCFAs produced by intestinal microbiota promoted IL-10 production (Sun et al., 2018), while binding of SCFAs to FFAR2 on colonic epithelial cells alle*via*ted colonic inflammation (Macia et al., 2015).

The metabolic consequences of FFAR2 activation can also be attributed to effects on adipocytes. SCFAs increase leptin secretion *via* FFAR2 both *in vitro* in adipocytes and *in vivo* in mice (Zaibi et al., 2010). In a different study, FFAR2 signaling inhibited lipogenesis in adipose tissue (Kimura et al., 2013). Together, this data suggest a possible role for the FFAR2 signaling pathway in preventing the deleterious consequences of metabolic syndrome and subsequent NAFLD.

FFAR2 stimulates enteroendocrine L cell GLP-1 secretion and prevents hyperglycemia. FFAR2 knockout mice have reduced GLP-1 secretion and impaired glucose tolerance compared to wild-type mice (Tolhurst et al., 2012). In a murine NAFLD model, the SCFA sodium butyrate prevented progression of NAFLD to NASH and increased GLP-1 receptor expression (Zhou et al., 2018). However, the authors concluded that sodium butyrate may prevent fatty liver disease progression *via* histone deacetylase rather than FFAR2 as GLP-1 receptor expression was unaffected by FFAR2 siRNA *in vitro*.

FFAR2 is structurally similar to FFAR3 which has made the development of selective FFAR2 agonists challenging. Compounds 1 and 2 have been developed as potent and selective FFAR2 agonists (Hudson et al., 2013). Relative to FFAR1 and FFAR4, the hepatic effects of FFAR2 signaling are indirect as FFAR2 is not expressed in the liver. Research is ongoing to determine if FFAR2-selective agonists can ameliorate metabolic liver disease and other disorders of dysregulated energy homeostasis (Milligan et al., 2017).

## FFAR3/GPR41

FFAR3 shares ~40% amino acid homology with FFAR2 and is conserved among species (Brown et al., 2003; Wang et al., 2009). FFAR3 is expressed in sympathetic ganglion, enteroendocrine, and pancreatic β-cells (Kimura et al., 2011; Priyadarshini and Layden, 2015). However, conflicting data exist as to whether or not FFAR3 is expressed in adipocytes (Mishra et al., 2020). Similar to FFAR2, hepatic expression of FFAR3 has not been reported. FFAR3 plays an important role in neural activity, lipid metabolism, and regulation of plasma glucose (Kimura et al., 2011). Similar to FFAR2, FFAR3 also preferentially binds SCFAs (Brown et al., 2003). However, FFAR3 potency is greatest with SCFAs of 3–5 carbon length (Le Poul et al., 2003). FFAR3 effects are mediated through Gi/o proteins and result in cAMP inhibition and phosphorylation of ERK1/2 (Brown et al., 2003).

FFAR3 activation, similar to FFAR2, stimulates GLP-1 secretion, thereby preventing hyperglycemia (Tolhurst et al., 2012). Dietary intake of SCFAs in mice protects against HFD-induced obesity and improves hepatic lipid metabolism (Shimizu et al., 2019). In FFAR3 deficient-mice, SCFA administration does not protect against HFD-induced obesity or steatosis (Shimizu et al., 2019). In a different study, FFAR3 knockout mice had reduced weight gain compared to wild-type mice under standard laboratory conditions but this difference was lost under germ-free conditions (i.e., in the setting of a reduced microbiome; Samuel et al., 2008). Collectively, these findings suggest that FFAR3 is essential for normal growth which is mediated in part by SCFA produced by intestinal flora. Furthermore, FFAR3 signaling may be a means to protect against NAFLD. However, the overlap in ligand binding and function between FFAR3 and FFAR2 could potentially confound these findings (Ichimura et al., 2014).

Development of selective FFAR3 ligands has been limited by the structural similarity between FFAR2 and FFAR3 and the low potency of endogenous ligands (Kimura et al., 2020). Selective FFAR3 agonists would be useful in research applications and potentially human therapeutics. Hudson et al. (2014) report on a potent positive allosteric modular-agonist of FFAR3, while other investigators have reported on the use of FFAR3 selective agonists such as AR420626 (Schmidt et al., 2011; Engelstoft et al., 2013). In continued similarity to FFAR2, FFAR3 is not expressed in the liver but FFAR3 signaling nonetheless poses potential to have significant though indirect hepatic effects. Future studies investigating FFAR3 agonists in the NAFLD/NASH models would be beneficial in further evaluating the therapeutic potential of these agents in liver disease.

## FFAR4/GPR120

FFAR4 is a GPCR for unsaturated LCFAs expressed in many human tissues (Hirasawa et al., 2005). Highest FFAR4 expression is found in lung, pituitary, small intestine, colon, adipose tissue, and macrophages including Kupffer cells (Gotoh et al., 2007; Oh et al., 2010). Two human FFAR4 isoforms exist: a short form of 361 residues and a long form of 377 residues. The long form has an interposed 16-residue segment in the third intracellular loop. Both isoforms are activated by omega-3 FAs. However, downstream signaling differs between isoforms. LCFA binding to the short isoform results in G-protein-dependent activation of phospholipase C and intracellular calcium mobilization (Watson et al., 2012). This is the primary mechanism for FFAR4-mediated hormone secretion. However, the interposed residues in the long isoform prevent G-protein signaling. Both the short and long isoforms bind β-arrestin 2 through a G-protein-independent mechanism and are internalized (Watson et al., 2012). The FFAR4/β-arrestin 2 complex inhibits inflammatory cascades. Interaction of the FFAR4/β-arrestin 2 complex with NLRP3 (nucleotide-binding oligomerization leucine-rich repeat and pyrin domain-containing protein 3) (NLRP3) prevents Caspase-1 from cleaving pro-IL-1β and pro-IL-18 into IL-1β and IL-18, respectively, (Yan et al., 2013). FFAR4/β-arrestin 2 also interacts with TAK1-binding protein 1 (TAB1), inhibiting TAK1 activation and stimulation of the IKKβ/NF*κ*B and JNK/AP1 pathways (Oh et al., 2010).

FFAR4 is a key mediator of hormone secretion in the gastrointestinal tract and pancreas. In the stomach and duodenum, activation of FFAR4 inhibits ghrelin secretion (Gong et al., 2014). FFAR4 activation in intestinal L- and K-cells results in secretion of GLP-1, GIP, inhibition of glucagon-like-peptide 2 (GLP-2), and CCK (Hirasawa et al., 2005; Iakoubov et al., 2007; Tanaka et al., 2008; Tsukahara et al., 2015). However, GLP-1 secretion may be more dependent on FFAR1 rather than FFAR4 activation (Xiong et al., 2013). In mouse pancreatic islets, ligand binding inhibits *δ*-cell somatostatin secretion and stimulates β-cell insulin secretion (Zhang et al., 2017). In adipose tissue, FFAR4 mediates adipocyte differentiation and enhances glucose uptake *via* GLUT4 translocation (Oh et al., 2010). Anti-inflammatory effects have been noted in macrophages and across a range of tissues including adipose tissue, skeletal muscle, liver, and brain (Oh et al., 2010; Raptis et al., 2014; Wellhauser and Belsham, 2014; Williams-Bey et al., 2014).

Hepatic expression of FFAR4 is primarily found in Kupffer cells. Emerging evidence suggest that the potent anti-inflammatory effects of FFAR4 agonism, in addition to the systemic metabolic effects, may protect against liver injury. Obese children carrying the R270H loss-of-function mutation are more likely to have elevated alanine aminotransferase (ALT) level and higher ferritin, suggesting increased liver injury and inflammation (Marzuillo et al., 2014). Another FFAR4 variant (rs11187533) has been associated with lower fasting glucose and decreased markers of liver injury in obese children (Codoner-Alejos et al., 2020).

FFAR4 holds particular relevance in metabolic and inflammatory disorders which have subsequent harmful effects on the liver. Adipocyte differentiation and lipogenesis are FFAR4-dependent. FFAR4 knockout mice fed a HFD had increased weight gain, adiposity, hepatosteatosis, fasting plasma glucose, and insulin resistance compared to wild-type mice (Ichimura et al., 2012). In addition, obese humans were found to have increased FFAR4 expression in adipose tissue. A FFAR4 loss of function mutation (p.R270H) is associated with risk of obesity. Inflammatory conditions are a key component in the pathogenesis of insulin resistance and metabolic syndrome and have been linked to FFAR4. A synthetic, specific, small molecule FFAR4 agonist (cpdA) administered to mice receiving a HFD prevented adipocyte tissue macrophage infiltration, decreased expression of the pro-inflammatory genes including TNF-α, IL-1β, and IL-6, increased insulin sensitivity, and decreased hepatosteatosis (Oh et al., 2014). These effects were not seen in FFAR4 knockout mice. In addition, pre-treatment of macrophages *in vitro* with DHA or cpdA prevented lipopolysaccharide-induced activation of the TAK1, IKKβ/NFκB, and JNK/AP1 pathways.

Omega-3 FAs are FFAR agonists and have been investigated in preventing hepatic ischemia–reperfusion injury. Injection of the intravenous omega-3 fatty acid rich fish oil lipid emulsion Omegaven® 1 h prior to murine hepatic ischemia decreased subsequent ALT, aspartate aminotransferase (AST), and hepatic necrosis (Raptis et al., 2014). In the same study, Omegaven® administered *in vitro* to hepatic macrophages resulted in FFAR4 activation and internalization similar to a FFAR4 agonist. In a murine model of parenteral nutrition-induced liver injury, intravenous fish oil was hepatoprotective in wild-type mice, but not in FFAR4 knockout mice (Fell et al., 2019). However, Baker et al. (2019) demonstrated that intravenous fish oil administration prior to hepatic ischemia–reperfusion injury in FFAR4 knockout mice protected against injury, suggesting that FFAR4 alone does not account for the hepatoprotective effects of fish oil.

Administration of a FFAR4 agonist in a murine model of NASH suppressed macrophage infiltration and reversed hepatic inflammation (Chen et al., 2020). Similarly, DHA prevented development of steatohepatitis in a murine model of NASH in wild-type mice, but not in FFAR4 knockout mice (Nakamoto et al., 2018). In 20 children with NAFLD treated with DHA for 18 months, no changes were observed in BMI, γ-glutamyl transferase (GGT), or basal insulin and glucose (Nobili et al., 2014). However, the significant decreases were seen in triglycerides, ALT, and AST. Liver biopsy (obtained before and after treatment) demonstrated DHA-associated marked reduction in steatosis, decreased inflammatory macrophages, and increased hepatocyte FFAR4 expression. Two randomized controlled trials using DHA supplementation in children with ultrasound-proven NAFLD have demonstrated improved insulin resistance and reduced hepatic steatosis (Nobili et al., 2011, 2013). An additional randomized controlled trial found that omega-3 FA supplementation did not reduce ALT or steatosis on ultrasound, but did decrease AST and GGT, while increasing adiponectin (Janczyk et al., 2015).

Omega-3 FA supplementation has also been studied in the models of alcoholic liver disease. Ethanol induces adipocyte death, FA release, and altered adipokine secretion leading to hepatosteatosis (Zhong et al., 2012). Omega-3 FAs prevent ethanol-induced adipose lipolysis through FFAR4 stimulation and decrease hepatotoxicity and steatosis (Wang et al., 2017). As mentioned previously, omega-3 FAs are not FFAR-specific agonists. More studies are required to establish that omega-3 FA-induced improvements in liver disease are mediated through FFAR4-dependent pathways.

Targeting FFAR4 as a therapy to modulate metabolic and inflammatory pathways is under active investigation. In addition to omega-3 FA supplementation, numerous small molecule agonists have been studied: GW9508, TUG-891, NCG21, Merck cpdA, GSK137647A, GSK cpd39, GPR120 agonist III, and PBI-4547 (Ulven and Christiansen, 2015; Chen et al., 2020; Simard et al., 2020). A key challenge remains the suboptimal selection of FFAR4 over FFAR1 among many agonists. A FFAR4-selective small molecule holds significant promise for the treatment of metabolic and inflammatory disease, including NAFLD, alcoholic liver disease, and other liver pathologies.

## Discussion

Free fatty acid receptors are a recently discovered class of GPCRs that are responsible for many integral agonist- and tissue-specific responses to dietary FAs. In states of health, FFAR signaling promotes GSIS, enterohepatic and enteroendocrine homeostasis, and nutrient-sensitive energy regulation, in addition to a host of other extra-intestinal effects. Many animal studies and human clinical trials have demonstrated the potential of naturally occurring and synthetic FFAR agonists in treating diabetes. More recently, omega-3 fatty acids, which are known agonists of FFAR1 and FFAR4, have shown efficacy in treating NAFLD, NASH, alcoholic hepatitis, and IFALD. While the hepatoprotective effects of omega-3 fatty acids may be mediated through non-FFAR pathways, evidence is mounting to suggest that FFARs are central regulators of hepatic energy metabolism. Further experiments using FFAR knockout animals and/or FFAR-specific antagonists in the liver disease models could provide insight into the mechanism of these diseases, determine if omega-3 FA hepatoprotection is mediated through FFAR-dependent pathways, and further characterize FFARs as clinical targets for liver disease.

The deleterious hepatic effects of altered or absent FFAR1 and FFAR4 signaling have been independently demonstrated through the knockout animal models, mutant alleles, and receptor antagonists. Furthermore, the expression of FFAR1 on hepatocytes and FFAR4 on hepatic Kupffer cells makes targeting these receptors as an intuitive strategy for treating liver disease. Experimental evidence suggest that FFAR-targeted therapies offer the potential to reduce hyperglycemia without inducing hypoglycemia, promote insulin sensitivity, reduce obesity, and prevent hepatic lipotoxicity. Agents with this capacity would benefit the hundreds of millions of patients worldwide with overweight, obesity, T2DM, and/or NAFLD. Furthermore, FFAR agonists with similar effects could potentially benefit patients with IFALD and other metabolic liver diseases. As of yet, no one FFAR agonist has demonstrated this potential but many agents currently under investigation have shown promise.

In summary, leveraging FFAR signaling to treat metabolic liver disease is a potential area of research that has largely been unexplored. While many studies have focused on the insulin-sensitizing effects of FFAR agonism, more investigation is needed into the direct and indirect hepatic effects of FFAR agonists. Specifically, examining the effects of natural and synthetic FFAR1- and FFAR4-specific agonists in treating NAFLD, NASH, and IFALD are promising avenues that warrant further investigation.

## Author Contributions

JS contributed to the conception, outlining, writing, editing, and formatting of the review. SF and ST contributed writing and editing. LY created and edited the figure. MP contributed to the conception, supervision, and editing. All authors contributed to the article and approved the submitted version.

### Conflict of Interest

MP is a consultant for Pronova/BASF, NorthSea Therapeutics, and has received research support from NorthSea Therapeutics, Otsuka Pharmaceutical Company, Alcresta, and Fresenius Kabi. Patent/Royalties for Omegaven are forthcoming. The authors have no other relevant affiliations or financial involvement with any organization or entity with a financial interest in or financial conflict with the subject matter or materials discussed in the manuscript. This includes employment, consultancies, honoraria, stock ownership or options, expert testimony, grants or patents received or pending, or royalties.
